# Impact of left ventricular diastolic function and survival in patients with severe aortic stenosis undergoing transcatheter aortic valve replacement

**DOI:** 10.1371/journal.pone.0196031

**Published:** 2018-05-02

**Authors:** Kimi Sato, Serge Harb, Arnav Kumar, Samir R. Kapadia, Stephanie Mick, Amar Krishnaswamy, Milind Y. Desai, Brian P. Griffin, L. Leonardo Rodriguez, E. Murat Tuzcu, Lars G. Svensson, Zoran B. Popović

**Affiliations:** Heart and Vascular Institute, Cleveland Clinic, Cleveland, Ohio, United States of America; Scuola Superiore Sant’Anna, ITALY

## Abstract

In year 2016, the American Society of Echocardiography (ASE) and the European Association of Cardiovascular Imaging (EACVI) updated Recommendations for the assessment and grading of diastolic dysfunction (DD). We aimed to assess the applicability of this DD grading method and its association with prognosis in patients with severe aortic stenosis (AS) who underwent transcatheter aortic valve implantation (TAVI). We retrospectively identified 237 consecutive patients with severe AS who underwent trans-femoral TAVI. Baseline transthoracic echocardiography was evaluated to assess pre- and post-TAVI diastolic function according to the current ASE/EACVI Recommendations. Prior to TAVI, 41 (17%) patients were diagnosed as having grade I DD, 111 (47%) patients had grade II DD, 80 (34%) had grade III DD. DD grade after TAVI decreased (p < 0.001) with 75 patients (32%) reclassified to a lower DD grade. During the median follow-up of 1,320 days, 136 (57%) patients died. In univariable Cox proportional hazards model analysis, neither pre- nor post-TAVI DD grade were associated with prognosis. However, patients with grade III DD detected before TAVI and AR≥ 2 after TAVI had poorer survival (p<0.008). Patients with grade III DD detected after TAVI and AR≥ 2 after TAVI had poorer prognosis (p = 0.002). TAVI improves DD grade. While poor DD grade was not associated with mortality after treatment of AS by TAVI, concomitant presence of DD and post-procedural AR carried a poor prognosis.

## Introduction

Since the advent of transcatheter aortic valve implantation (TAVI), it has become clear that there is a large population of elderly patients with aortic stenosis (AS) who were underserved in previous decades, as they were deemed inoperable or very high surgical risk [1]. These patients virtually always have significant accompanying cardiovascular abnormalities, including abnormalities of diastolic function. On the other hand, the very high risk nature of their condition, combined with associated frailty, makes invasive diagnosis of diastolic function abnormalities an unappealing choice. Therefore, there is an emerging need to have a reliable non-invasive method in these patients to detect abnormalities of diastolic function, with or without concomitant presence of elevated filling pressures.

Echocardiographic assessment of diastolic function is a cornerstone of non-invasive diastolic function assessment. It relies on measuring combinations of echo parameters that are then subjected to a grading algorithm, with the end result of categorizing the patients into incremental grades of diastolic dysfunction (DD). Recently, the American Society of Echocardiography (ASE) and the European Association of Cardiovascular Imaging (EACVI) proposed 2016 Recommendations for the assessment and grading of diastolic function which appear to be superior, albeit modestly, to previously proposed methods [2,3]. Yet these recommendations potentially suffer from application in the TAVI patient population.

This population often shares several distinct features. Elderly patients, who constitute the vast majority of the TAVI population, by nature, have worse diastolic function parameters that are a consequence of non-pathologic aging. In addition TAVI patients often have atrial fibrillation (AF), or history of AF, abnormalities of conduction, and/or pacemaker implantation, which all can affect presence of atrial contraction which is a necessary element for the application of the Recommendations grading algorithm [2]. TAVI patients, besides having AS -associated left ventricular hypertrophy (LVH) and mild /moderate aortic regurgitation (AR), almost always have at least some mitral valve pathology such as severe mitral annular calcification (MAC) accompanied by at least some mitral stenosis (MS) or mitral regurgitation (MR) [4] [5,6,7,8,9]. They also frequently have coronary artery disease. Finally, the recent Recommendations cover the isolated presence of AF or mitral valve pathology in the “special condition” recommendations and suggest additive use of several unique variables to predict elevated LV filing pressure. However, these additional recommendations are incomplete as they do not propose specific cut-off values for each DD grade, and deal with only one special condition at a time, instead of a constellation of several conditions, as is almost invariably seen in patients undergoing TAVI [2].

We devised this retrospective study to answer the following questions: 1) How often can we apply the 2016 ASE/EACVI Recommendations to the TAVI population? 2) Can we apply DD grading in this population even if special conditions, such as AF or mitral valve abnormality, are present? and 3) Does DD grade defined by the 2016 Recommendations correlate with prognosis?

## Materials and methods

### Study sample

We retrospectively identified consecutive patients with severe AS who underwent trans-femoral TAVI with an Edwards SAPIEN and SAPIEN-XT balloon-expandable bioprosthetic valve (Edwards Lifesciences, Irvine, CA) at Cleveland Clinic between May 2006 and December 2012. Patients with no pre-TAVI echocardiographic assessment or mitral valve surgery were excluded. The baseline echocardiogram was evaluated to obtain diastolic function according to updated ASE/EACVI DD Recommendations. In addition, if patients had a transthoracic echocardiographic assessment followed by a TAVI procedure within 24 hours, the association between hemodynamic variables and DD grade was evaluated. Clinical and demographic data were obtained via manual extraction from electronic medical records. The survival status of all patients after TAVI was also collected. We followed patients by chart review with date of last follow-up or death recorded. In addition, we obtained mortality data from medical records or state and nationally available databases and internet sources (last queried April 2017). We used all-cause mortality as the primary outcome. The study was approved by the Cleveland Clinic Institutional Review Board and informed consent was waived as it was a retrospective study. All data were fully anonymized prior to the analyses.

### Echocardiographic measurement

Comprehensive transthoracic echocardiograms were available in all 237 patients within three month (median 1 day, range 1 to 43 days) before TAVI, and in 235 patients within one month (median 2 days, range 2 to 7 days) after TAVI. All studied echocardiographic parameters were re-analyzed offline by an independent investigator (KS) blinded to hemodynamic and data according to guidelines. [2,10,11,12,13,14] DD grade was decided according to these measurements. Parameters measured included left atrial volume index (LAVi), mitral LV inflow peak early (E) and late diastolic (A) velocities and A wave duration, isovolumic relaxation time (IVRT), septal and lateral mitral annular e’ velocities, pulmonary venous flow peak systolic (S) and peak anterograde diastolic (D) velocities, and peak tricuspid regurgitation (TR) velocity. Where possible, each measurement was averaged over multiple cardiac cycles. Of note, in patients with AF, measurements were averaged from 3 nonconsecutive beats with cycle lengths within 10% to 20% of the average heart rate. [15] MAC severity was assessed by the combined use of visual assessment and Doppler measurement. Severe MAC was defined as severe calcification of the mitral annulus or moderate calcification with mitral valve mean pressure gradient higher than 3 mmHg. Aortic valve area (AVA) was calculated by the 2D-Doppler method using the continuity equation. The severity of aortic regurgitation (AR) after TAVI was assessed by transthoracic echocardiography within 30 days of the procedure. [11,12] The severity of AR was graded on a clinical scale ranging from “none” to “4” using following information; left ventricular size, the regurgitant jet was evaluated for flow convergence, vena contracta, and jet area at a Nyquist limit of 50 to 60 cm/s in parasternal long-axis and apical views; the circumferential extent of the regurgitant jet was evaluated in the parasternal short-axis view; jet density and pressure halftime were evaluated by continuous-wave Doppler in the best aligned views; and diastolic flow reversal was evaluated by pulse-wave Doppler in the descending thoracic aorta.

### Diastolic function assessment

**Fig 1:**
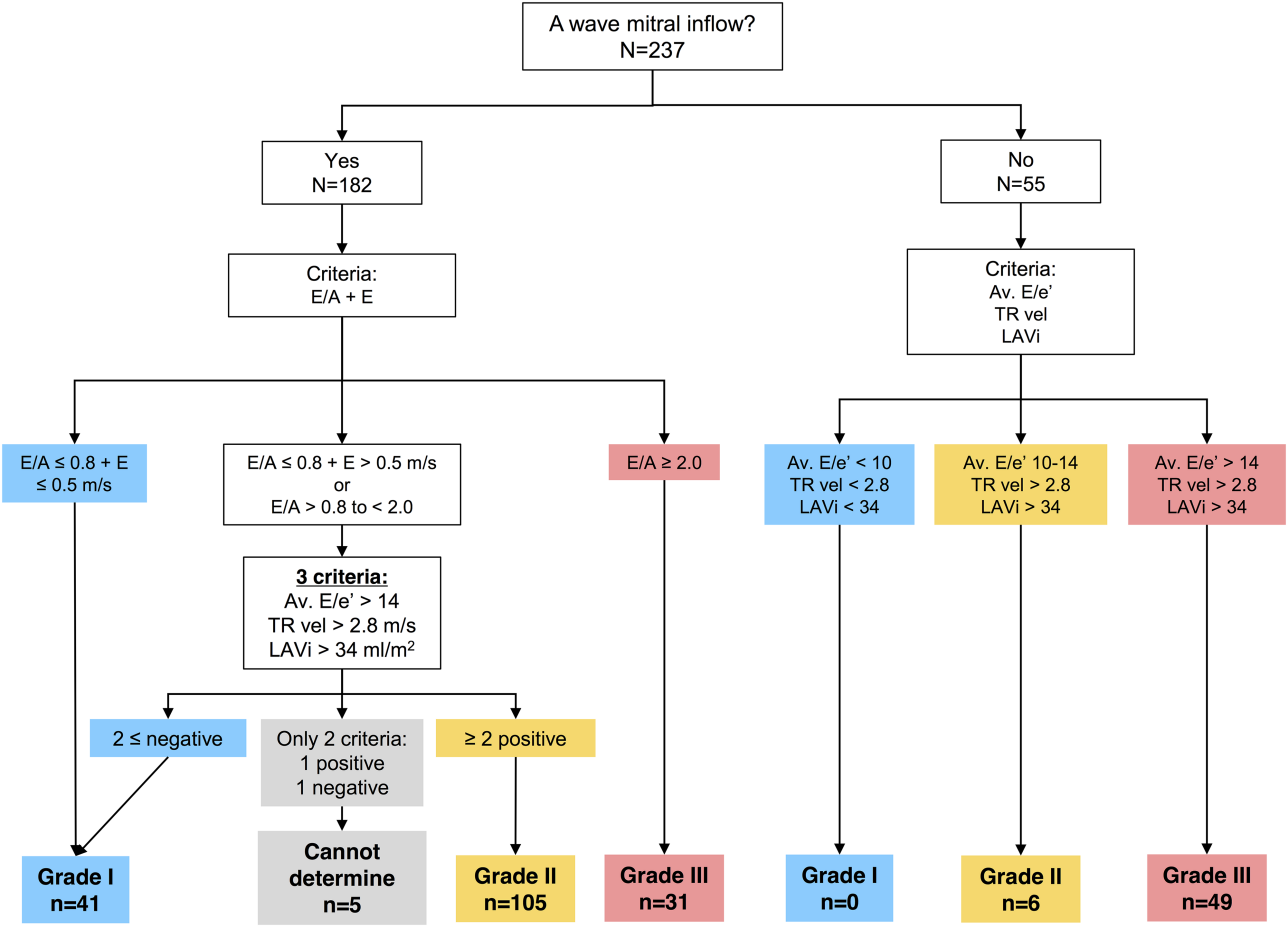
Flow diagram for the evaluation of diastolic dysfunction by ASE/EACVI 2016 Recommendations. Patients were classified as grade I, II, or III diastolic dysfunction or “cannot determine” category. Abbreviations: TR, tricuspid regurgitation; LAVi, left atrial volume index; Av E/e’, ratio between mitral inflow E velocity and the average of septal and lateral mitral e’.

Pre-TAVI and post-TAVI LV diastolic function grade was assessed according to ASE/EACVI Recommendations (Fig 1) [2]. Grading was implemented exclusively based on measurements using a software-based algorithm, without any operator interaction. The ASE/EACVI Recommendations propose a DD decision making algorithm composed of a two-step decision flowchart, with a first-step flowchart used to triage for the presence of DD in patients with normal LVEF, with all other patients proceeding directly to step two. In addition, a figure legend that accompanies the flowcharts mentions that normal LVEF patients with “myocardial disease” should be assumed to have DD by default. As all of our patients had chronic long standing severe AS (and can be expected to have “myocardial disease” [16]), we directly applied the second-step flowchart to all. When only 2 parameters were available and only one parameter met the cutoff values or there was only one available parameter, patients with pulmonary vein S/D ratio < 1 and reduced EF were graded as grade 2, while the remaining patients were classified as having DD with “cannot determine” grade [2].

Although ASE/EACVI Recommendations suggested use of additional echo variables and careful assessment in patients with specific cardiovascular diseases, they do not propose an alternate algorithm or threshold to assess DD grading. Therefore, DD grading in patients with mitral valve abnormalities were assessed using the same algorithm if their E/A ratio could be analyzed. On the other hand, in patients whose E/A ratio could not be analyzed due to AF, ventricular pacing, or inadequate image quality, the decision making algorithm was not applicable. Hence, we assessed DD grade using three variables (average E/e’, TR velocity, LAVi) using cut-off values proposed in Table 4 of a published 2016 Recommendations text (see Fig 1) [2]. In cases where variable values did not fall in the same DD grade, DD grade was established as that with the highest number of parameter values characteristic to that grade [2], assuming equal weighting. For example, a patient with AF, E/e’ >14, and LAVi>34, but with TR velocity <2.8 m/s would fall into the Grade III category.

### Cardiac catheterization

To assess the relationship between echocardiographic DD grade and invasive hemodynamics, we assessed the data of 50 patients who had echocardiographic evaluation within 24 hours prior to catheterization. Hemodynamic evaluation was performed during the procedure before the prosthetic valve deployment by a standard transfemoral approach. LV pressure was recorded using a fluid-filled catheter. Hemodynamic tracings were retrieved and re-evaluated to obtain LV end-diastolic pressure (LVEDP).

### Statistical analysis

Continuous data are expressed as mean ± standard deviation when normally distributed, or median (interquartile range). Categorical data are presented as absolute numbers and percentages. We used the paired t test, Wilcoxon signed-rank test, McNemar’s test, and Kruskal-Wallis test to compare the data between two-groups or more as appropriate. Previous data suggested that presence of post-operative AR modulates the impact of diastolic function on survival. To assess for this potential interaction between DD grade and post-procedural AR,[17] we defined 4 groups as follows: DD grade ≤II/AR<2; DD grade ≤II/AR≥2; DD grade III/ AR<2; and DD grade III/AR≥2. Unadjusted survival curves were constructed by the Kaplan Meier method. We assessed differences in survival between the groups using log rank statistics. This was followed by univariable and multivariable Cox proportional hazards model analysis. In the multivariable model, possible confounders (e.g., STS score) were entered into the model with forward stepwise selection. A P value of < 0.05 was considered statistically significant. We performed statistical analyses with JMP 10.0 (SAS Institute Inc., Cary, NC), and SPSS 23.0 software (SPSS Inc., Chicago. IL).

## Results

The final group consisted of 237 consecutive patients with severe AS who satisfied inclusion criteria. Patients’ baseline demographics and echocardiographic variables are shown in Table 1. At baseline, AF was observed in 37 patients (16%) and severe MAC was present in 30% of them. The feasibility of obtaining echocardiographic parameters recommended by the ASE/EACVI Recommendations was more than 90% except for E/A which could be obtained in 77% of cases.

**Table 1:** Patient baseline characteristics.

	n = 237
**Age (years)**	80±10
**Male gender**	140 (59%)
**Weight**	81.0±20.0
**Height**	167.9±10.9
**Body mass index (kg/m**^**2**^**)**	29.8±5.5
**NYHA (III/IV)**	195/28 (82/12%)
**Coronary artery disease**	200 (84%)
**STS score (risk of mortality)**	9.6±5.2
**Creatinine (mg/dl)**	1.16±0.46
**Atrial fibrillation**	37 (16%)
**Severe mitral annular calcification**	70 (30%)
**Pacemaker/ ICD**	23 (8%)

Values are mean ± SD or n (%). Abbreviations: NYHA, New York Heart Association Functional Classification; STS, Society of Thoracic Surgeons; ICD, implantable cardioverter defibrillator.

### Pre-TAVI detection of diastolic dysfunction in patients with normal EF by 2016 ASE/EACVI Recommendations

While the ASE/EACVI Recommendations provide first flowchart to triage for the presence of DD in patient with normal EF, it also suggests that all patients with “myocardial disease” should proceed directly to the second flowchart [2]. As one can assume that all patients undergoing TAVI for AS have myocardial disease and diastolic dysfunction [16], we assessed how well the first flowchart of the ASE/EACVI Recommendations detects the presence of DD in 134 patients with normal EF prior to TAVI. Among 134 patients, only 8% were diagnosed as having “normal” diastolic function, 20% were “indeterminate,” and 72% were diagnosed as having DD.

### Pre-TAVI grading of diastolic dysfunction by 2016 ASE/EACVI DD Recommendations

**Fig 2:**
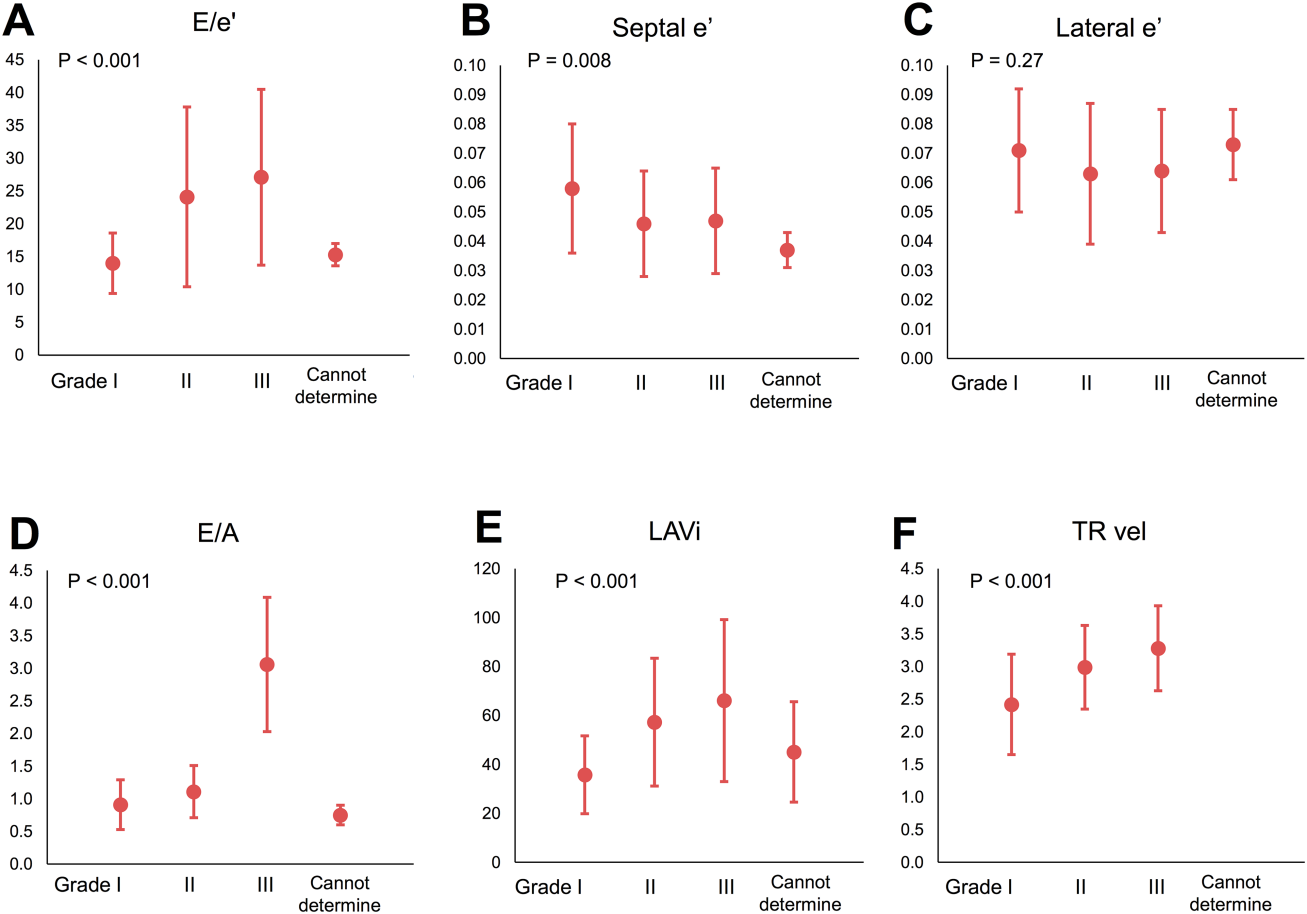
Association of diastolic function grading and echocardiographic variables proposed in Recommendations: A) E/e’, B) septal e’, C) lateral e’, D) E/A, E) LAVi, and F) TR velocity. Diastolic dysfunction grading significantly differed in E/e’, septale’, E/A, LAVi, TR velocity. Abbreviations: LAVi, left atrial volume index; TR, tricuspid regurgitation.

Since the first flowchart did categorize the vast majority of our patients with normal EF as having DD prior to TAVI, we directly applied a second flowchart to all pre-TAVI echocardiograms. In 55 patients in whom we were unable to calculate E/A, DD grading was decided using alternative criteria as described in the Methods and based on 2016 DD Recommendations. [2] We diagnosed grade I DD in 41 patients (17%), grade II in 111 (47%) patients, grade III in 80 (34%) patients, while 5 patients were classified as “cannot determine” DD (Fig 1, Table 2). DD grades differed in E/e’, septal e’, E/A, LAVi, and TR velocity, however with similar lateral e’ (Fig 2). When compared to expected values for the LV DD grade presented in Table 4 of the ASE/EACVI DD Recommendations [2], our patients had similar values for E/A ratio, LAVi, and TR velocity, but higher values of E/e’.

**Table 2:** Echocardiographic variables stratified by new ASE/EACVI Recommendations.

	All (N = 237)	Grade I (N = 41)	Grade II (N = 111)	Grade III (N = 80)	Cannot determine (N = 5)	P value
**MR > 2+**	30 (13%)	0	7 (6%)	23 (29%)	0	< 0.001
**Severe MAC**	70 (30%)	7 (17%)	43 (39%)	18 (23%)	2 (40%)	0.021
**MS > mild (n = 237)**	7 (3%)	2 (5%)	5 (5%)	0	0	0.26
**LVEF > 50% (n = 237)**	134 (57%)	29 (71%)	73 (66%)	27 (34%)	5 (100%)	< 0.001
**LVEF (%) (n = 237)**	49±14	53±13	53±12	42±15	62±3	< 0.001
**E/e’ (n = 220)**	23±13	14.0±4.6	24.1±13.7	27.1±13.4	15.3±1.7	< 0.001
**E’ septal (m/sec) (n = 221)**	0.05±0.02	0.06±0.02	0.05±0.02	0.05±0.02	0.04±0.01	0.008
**E’ lateral (m/sec)(n = 220)**	0.06±0.02	0.07±0.02	0.06±0.02	0.06±0.02	0.07±0.01	0.267
**LAVi (ml/m**^**2**^**) (n = 237)**	56±29	35.8±15.9	57.3±26.1	66.1±33.1	45.1±20.5	< 0.001
**TR velocity (m/sec) (n = 217)**	2.29±1.46	2.42±0.77	2.99±0.64	3.28±0.65	NA	< 0.001
**E/A (n = 182)**	1.40±0.95	0.91±0.38	1.11±0.40	3.06±1.03	0.75±0.15	< 0.001
**E (m/sec) (n = 235)**	1.16±0.38	0.88±0.31	1.18±0.40	1.30±0.30	0.85±0.10	< 0.001
**DT (msec) (n = 233)**	215±84	241±105	233±84	173±46	246±123	< 0.001
**S/D (n = 178)**	0.96±0.59	1.29±0.60	1.05±0.50	0.65±0.57	NA	< 0.001
**S/D < 1.0 (n = 178)**	106 (56%)	11 (37%)	46 (51%)	49 (86%)	0	< 0.001

Abbreviations: MR, mitral regurgitation; MAC, mitral annular calcification; MS, mitral stenosis; LVEF, left ventricular ejection fraction; LAVi, left atrial volume index; TR, tricuspid regurgitation; DT, deceleration time.

We additionally assessed the association between DD grading and hemodynamic parameters in 50 patients in whom hemodynamic assessment followed the echocardiogram within 24 hours. In this subgroup, 11 patients (22%) were diagnosed as having grade I DD, 22 (44%) had grade II DD, and 17 (34%) had grade III DD (S1 Table). The mean LVEDP was 21±7 mmHg, with all three DD groups having similar LVEDP (p = 0.52, rho 0.09) with a large overlap.

### Pre-TAVI LV diastolic function and subsequent survival

**Fig 3:**
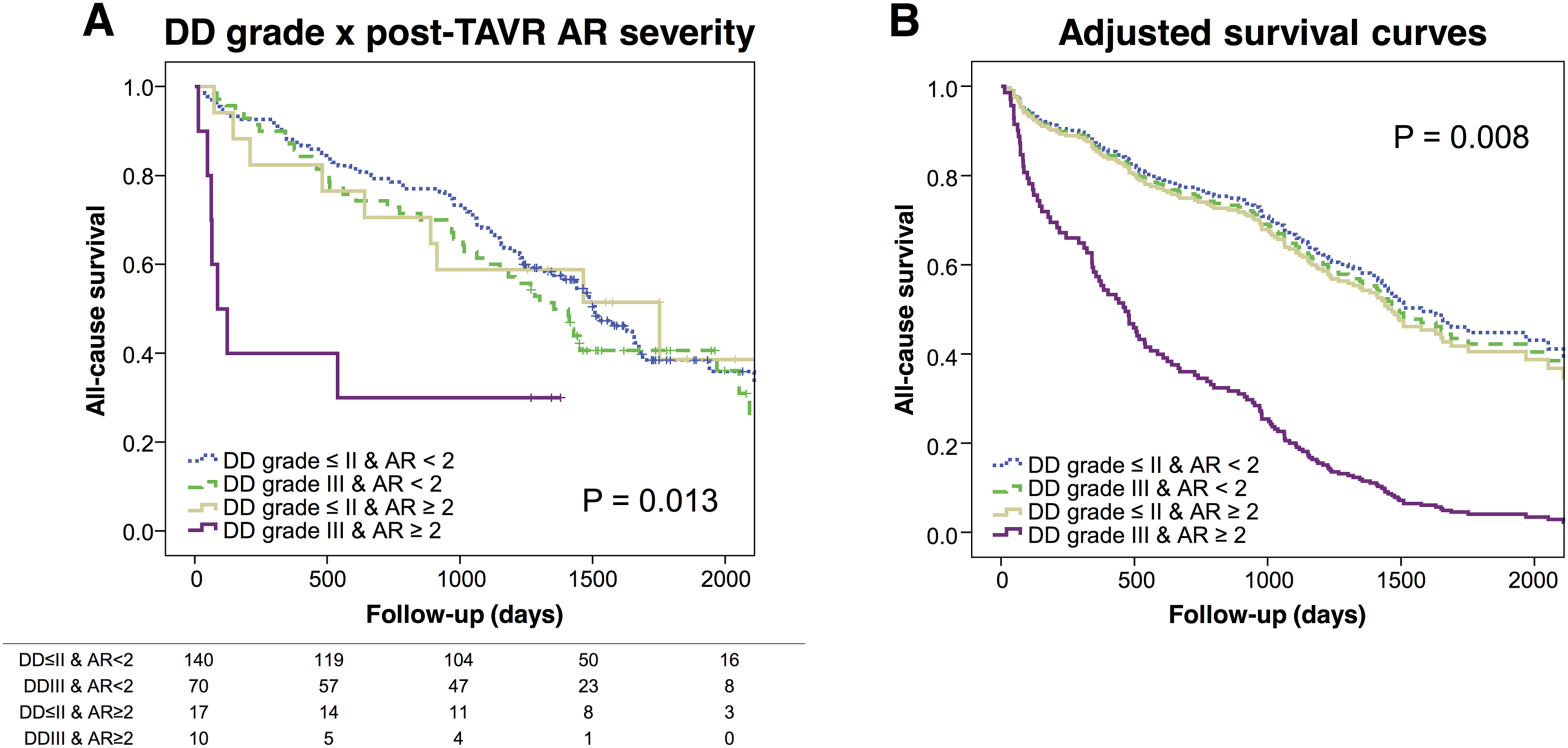
Survival curves with baseline diastolic dysfunction (DD) grade and post-procedural aortic regurgitation (AR) severity: A) Kaplan-Meier curves and B) adjusted survival curves for 4 groups of patients according to baseline DD (grade III or grade ≤ II) and post-procedural AR severity (AR ≥ 2 or < 2). Abbreviations: DD, diastolic dysfunction; AR, aortic regurgitation.

After TAVI, 27 patients (11%) developed at least moderate AR. Eighteen patients developed complete left bundle blanch block and 19 patients required pacemaker/ implantable cardioverter defibrillator implantation after TAVI. During a median follow-up of 1,320 days (interquartile range 733 to 1,618 days), 136 (57%) patients died with 34 patients (14%) were dying within first year. Death occurred in 21/41 (51%) patients classified as grade I DD, 62/111 (56%) patients classified as grade II, and 50/80 (63%) patients with grade III DD. As previously shown [18], baseline STS score (Hazard ratio [HR] 1.05, 95%CI: 1.01–1.08, p = 0.004) and post-procedural AR (HR 1.34, 95%CI: 1.02–1.75, p = 0.035) were associated with poor prognosis both in uni- and in multi-variable Cox proportional hazard models. In contrast, advanced DD grade and echocardiographic parameters used for DD grade determination according to ASE/EACVI DD Recommendations did not show significant association with overall mortality (S2 Table). When we repeated the analysis limited to 1-year mortality only, DD grade and other relevant echocardiographic variables again were not associated with survival. Only post-procedural AR was associated with poor 1-year survival in a multivariable model (HR 1.77, 95%CI: 1.78–4.55, p < 0.001). As a prior study showed that concomitant presence of severe DD can amplify the effects of post-procedural AR [17], we performed survival analysis after creating groups based on the presence of AR ≥2 and grade III DD (see Methods). The analysis showed that patients with concomitant presence of these factors were associated with poorer prognosis (Log-rank p = 0.013) (Fig 3A). These findings remained significant after adjusting for STS score (p = 0.008 by Cox proportional hazards) (Fig 3B).

### Post- TAVI LV diastolic function and subsequent survival

**Fig 4:**
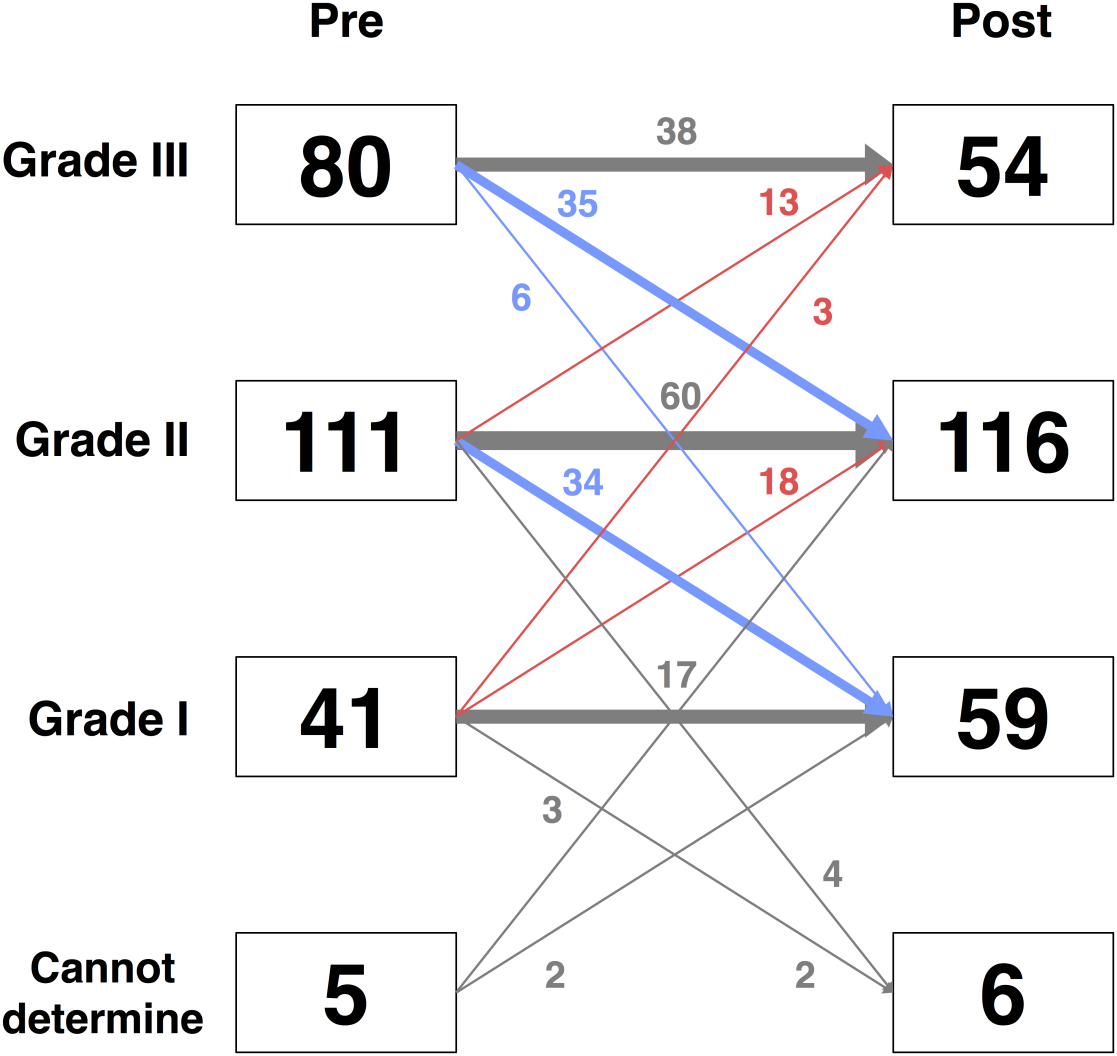
Reclassification of diastolic dysfunction (DD) grade after transcatheter aortic valve implantation (TAVI). Abbreviations: DD, diastolic dysfunction; TAVI, transcatheter aortic valve implantation.

**Fig 5:**
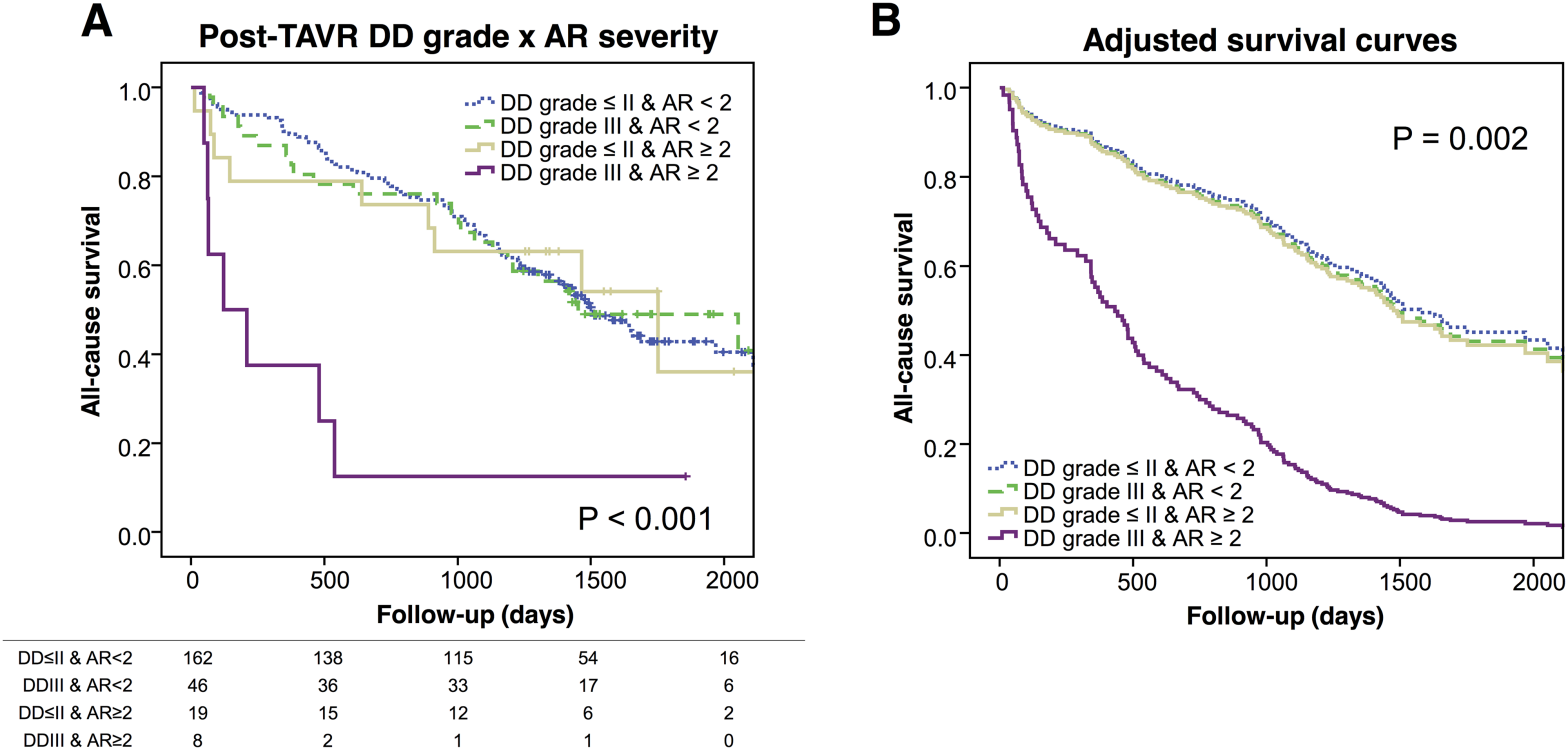
Survival curves with post-TAVI diastolic dysfunction (DD) grade and post-procedural aortic regurgitation (AR) severity: A) Kaplan-Meier curves and B) adjusted survival curves for 4 groups of patients according to baseline DD (grade III or grade ≤ II) and post-procedural AR severity (AR ≥ 2 or < 2). Abbreviations: DD, diastolic dysfunction; AR, aortic regurgitation.

S3 Table shows echocardiographic parameters at pre- and post-TAVI. TAVI improved AVA, mean aortic valve pressure gradient, E/e’, LAVi, and TR velocity. After TAVI, DD grade decreased (p < 0.001), with 59 patients (5%) having grade I DD, 116 (49%) grade II DD, and 54 (23%) grade III DD (Fig 4). Presence of new conduction abnormality, or AR, after TAVI were not associated with post-TAVI DD grade. In Cox proportional hazards model analysis, DD grading after TAVI was not associated with outcome (HR 1.16, 95%CI: 0.93–1.44, p = 0.20). However, similar to the pre-TAVI DD grading, concomitant presence of post-TAVI DD grade III and AR ≥ 2 was significantly associated with poorer prognosis (Log-rank p = < 0.001) (Fig 5). These findings remained significant after adjusting for STS score (p = 0.002 by Cox proportional hazards).

## Discussion

In the present study, we show that current ASE/EACVI DD Recommendations classify a majority of high risk patients with severe AS as having DD even in the setting of preserved EF. TAVI decreased DD grade. Interestingly we show that, while poor DD grades pre- and post- TAVI were not associated with higher mortality on their own, the concomitant presence of diastolic dysfunction and post-procedural AR was associated with a poor prognosis.

## Application of 2016 Recommendation in severe AS patients

The two factors that could influence DD grading in severe AS patients are absence of A wave on mitral inflow and MAC. We show that, absence of A wave could be circumvented if we apply alternate criteria to patients without measurable E/A ratio. Using these methods, 78% of our patients had pre-TAVI DD grade ≥ II, which is similar to prior reports. [17] Dealing with presence of MAC is more complex. Updated ASE/EACVI Recommendations note that application of the DD grading algorithm in patients with AS should pose no major challenge except for the presence of MAC. [2] However, the presence of some degree of MAC in our sample was ubiquitous, with 70/237 patients having severe MAC. [19,20,21,22,23,24] MAC and MAC-associated mitral valve disease in the TAVI population show a continuum of severity with large numbers of patients deemed to have “moderate” disease, thus making dichotomization into “significant” and “nonsignificant” mitral valve pathology often impractical. As it is well known that mitral valve pathology can impact E/A and E/e’ measurements,[5,6,7,8,25,26] its presence may be a reason why we could not document a stronger relationship between LVEDP and its echocardiographic estimates. On the other hand, using the presence of MAC as an exclusion criterion would result in most of the patients being eliminated from DD grading.

### Diastolic dysfunction and survival after TAVI

In our study individual echocardiographic variables and DD grade were either not associated, or showed only a trend, with increased risk of mortality post TAVI. Prior studies have shown somewhat conflicting results. A recent small, single-center study of 90 TAVI patients that showed that DD grade by ASE/ EACVI Recommendations and LAVi were borderline one-year survival predictors. [27] On the other hand, a recent study of 195 patients reported the lack of predictive ability of DD grade by ASE/EACVI Recommendations in patients undergoing TAVI [17]. The possible reason for these observations is that grading DD in the TAVI population is difficult. As mitral valve pathology (i.e., mitral annular calcification, stenosis or regurgitation) or systolic dysfunction can affect echocardiographic DD parameters, the reliability of DD classification by echocardiography may be limited. In addition, senescence may exert its effects on diastolic function parameters through slowed relaxation [28] and concentric remodeling. Another possibility is that correction of AS by TAVI could mask the impact of pre-procedural DD. Finally, high risk patients undergoing TAVI are often with multiple comorbidities and the impact of DD on survival may be masked by cardiac or non-cardiac risk factors. Indeed, higher STS score at baseline, which is reflective of noncardiac comorbidities, was a significant predictor of post-TAVI survival.

One exception for the weak impact of DD on survival appears to be in the presence of post-TAVI AR. Kampaktsis et al. [17] have shown that post-procedural AR changes the impact of DD grade, with much poorer survival occurring in patients with concomitant severe DD and AR. While their findings in isolation can be deemed as only hypothesis generating, our data are supportive of their findings. We show that severe DD, detected either before or after TAVI, is associated with poor survival only if at least moderate post-procedural AR is present. This further strengthens the importance of preventing residual AR after a TAVI procedure. Of note, as patients with concomitant severe DD and AR have worse baseline characteristics, [17] one can suggest that their maladaptive or deconditioned status could be a reason of poorer survival. Further studies are necessary to assess whether DD grade is a valid prognostic parameter in the new era of TAVI devices with less post-procedural AR and application of TAVI to patients who are intermediate or low risk.

We show that advanced DD classified by ASE/EACVI Recommendations is associated with poor prognosis in patients who develop significant AR after TAVI. This suggests some utility of DD grade evaluation by this algorithm. On the other hand, we show that nearly 50% of patients with severe AS who underwent TAVI have cardiac pathology (MAC, loss of A wave) which makes the application of the DD grading algorithm less straightforward. In addition, DD grading proposed by ASE/EACVI Recommendations shows limited predictive accuracy of detecting elevated LV filling pressures in this patient population. Further studies are necessary to improve DD grading in severe AS patients.

### Limitations

This study was a retrospective, observational study conducted at a large tertiary referral center and thus might suffer from selection bias. We did not collect data as to the cause of death, which limits our ability to draw conclusion as to the mechanisms linking DD dysfunction and residual AR to all-cause mortality. In addition, we performed multiple comparisons and multivariable analyses on a relatively small sample size of 237 subjects, with death occurring in 136 patients. Further studies are needed to corroborate or refute these findings. In addition, hemodynamic data were obtained in 50 patients with non-simultaneous echocardiographic and hemodynamic assessment, while hemodynamic and echocardiographic data were acquired within 24 hours with no major change in cardiovascular medications.

## Conclusions

Our findings indicate that the 2016 ASE/EACVI DD Recommendations classified 95% of high risk patients with severe AS as having DD prior to TAVI, with 81% of them having moderate or severe DD. TAVI procedure decreased DD grade. While high DD grades were not associated with higher mortality after correction of AS by TAVI, the presence of both diastolic dysfunction and post-procedural AR was associated with a poor prognosis.

## Supporting information

S1 Table Invasive hemodynamic variables stratified by new ASE/EACVI Recommendations. (DOCX)

S2 Table Univariable and multivariable Cox proportional hazards model analysis to predict over-all mortality after TAVI. (DOCX)

S3 Table Echocardiographic parameters and DD grade before and after TAVI. (DOCX)
