# Supplementary material for: Impact of left ventricular diastolic function and survival in patients with severe aortic stenosis undergoing transcatheter aortic valve replacement
Source: PLoS One. 2018 May 2;13(5):e0196031. doi: 10.1371/journal.pone.0196031 (PMC5931627; doi:10.1371/journal.pone.0196031)
Supplement: S1 Table — (DOCX) [file pone.0196031.s001.docx]

**S1 Table. Invasive hemodynamic variables stratified by new ASE/EACVI Recommendations**

|  | **Grade I**  **(N=11)** | **Grade II**  **(N=22)** | **Grade III**  **(N=17)** | **P value** |
| --- | --- | --- | --- | --- |
| **MAC (+)** | 2 (18%) | 11 (50%) | 3 (18%) | 0.05 |
| **LVEDP (mmHg)** | 20.3±4.0 | 20.7±7.6 | 23.1±7.9 | 0.52 |
| **LVEDP > 16 mmHg** | 9 (82%) | 14 (64%) | 14 (82%) | 0.33 |

Abbreviations: MAC, mitral annular calcification; LVEDP, left ventricular end-diastolic pressure.
