# Supplementary material for: Impact of left ventricular diastolic function and survival in patients with severe aortic stenosis undergoing transcatheter aortic valve replacement
Source: PLoS One. 2018 May 2;13(5):e0196031. doi: 10.1371/journal.pone.0196031 (PMC5931627; doi:10.1371/journal.pone.0196031)
Supplement: S2 Table — (DOCX) [file pone.0196031.s002.docx]

**S2 Table. Univariable and Multivariable Cox Proportional Hazards Model Analysis to Predict Over-all Mortality after TAVI**

|  | **Univariable** | | **Multivariable** | |
| --- | --- | --- | --- | --- |
|  | **HR (95%CI)** | **P value** | **HR (95%CI)** | **P value** |
| **STS score** | **1.04 (1.01-1.07)** | **0.011** | **1.05 (1.01-1.08)** | **0.007** |
| **E/e’ average** | **1.01 (0.99-1.02)** | **0.26** |  |  |
| **LAVi** | **1.00 (0.99-1.01)** | **0.16** |  |  |
| **TR velocity** | **1.00 (0.99-1.00)** | **0.36** |  |  |
| **E/A** | **1.02 (0.80-1.26)** | **0.87** |  |  |
| **LVEF** | **0.99 (0.99-1.01)** | **0.87** |  |  |
| **Severe MAC** | **1.28 (0.88-1.83)** | **0.19** |  |  |
| **Post-TAVI AR** | **1.36 (1.03-1.79)** | **0.032** | **1.44 (1.07-1.93)** | **0.018** |
| **Post-TAVI CLBBB** | **0.83 (0.58-1.98)** | **0.84** |  |  |
| **Post-TAVI device implantation** | **1.15 (0.63-2.08)** | **0.65** |  |  |
| **MR >=moderate** | **0.89 (0.51-1.46)** | **0.66** |  |  |
| **DD grade** | **1.16 (0.91-1.49)** | **0.23** |  |  |

Abbreviations: TAVI, transcatheter aortic valve implantation; HR, hazards ratio; CI, confidence interval; STS, Society of Thoracic Surgeons; LAVi, left atrial volume index; TR, tricuspid regurgitation; EF, ejection fraction, MAC, mitral annular calcification, AR, aortic regurgitation; CLBBB, complete left bundle branch block; MR, mitral regurgitation, DD, diastolic dysfunction.
