# Supplementary material for: Impact of left ventricular diastolic function and survival in patients with severe aortic stenosis undergoing transcatheter aortic valve replacement
Source: PLoS One. 2018 May 2;13(5):e0196031. doi: 10.1371/journal.pone.0196031 (PMC5931627; doi:10.1371/journal.pone.0196031)
Supplement: S3 Table — (DOCX) [file pone.0196031.s003.docx]

**S3 Table. Echocardiographic Parameters and DD Grade Before and After TAVI**

|  | **No.** | **Pre TAVI** | **No.** | **Post TAVI** | **p value** |
| --- | --- | --- | --- | --- | --- |
| **Echocardiographic parameters** |  |  |  |  |  |
| AVA (cm^2^) | 237 | 0.70±0.24 | 208 | 1.42±0.38 | < 0.001 |
| Peak velocity (m/sec) | 237 | 4.33±0.79 | 215 | 2.13±0.39 | < 0.001 |
| AV mean PG (mmHg) | 237 | 46±16 | 215 | 10±4 | < 0.001 |
| AR (≥moderate) | 237 | 45 (19 %) | 237 | 27 (11 %) | 0.49 |
| LVEDV (ml) | 237 | 113±49 | 189 | 100±43 | <0.001 |
| LVESV (ml) | 237 | 60±43 | 221 | 49±36 | <0.001 |
| LVEF (%) | 237 | 49±15 | 233 | 49±13 | 0.78 |
| **DD parameters** |  |  |  |  |  |
| E/e’ average | 220 | 23±12 | 200 | 21±9 | < 0.001 |
| LAVi | 237 | 56±29 | 228 | 46±17 | < 0.001 |
| TR velocity | 217 | 3.00±72 | 225 | 2.81±74 | < 0.001 |
| E/A | 182 | 1.39±0.94 | 179 | 1.35±0.83 | 0.14 |
| **DD grade** | 237 |  | 235 |  | < 0.001 |
| Grade I |  | 41 |  | 59 |  |
| Grade II |  | 111 |  | 116 |  |
| Grade III |  | 80 |  | 54 |  |
| Cannot determine |  | 5 |  | 6 |  |

Values are mean ± SD or n (%). Abbreviations: TAVI, transcatheter aortic valve implantation; AVA, aortic valve area; AV, aortic valve; PG, pressure gradient; AR, aortic regurgitation; LVEDV, left ventricular end-diastolic volume; LVESV, left ventricular end-systolic volume; LVEF, left ventricular ejection fraction; DD, diastolic dysfunction; LAVi, left atrial volume index; TR, tricuspid regurgitation.
